# Analytical Performance of Clay Paste Electrode and Graphene Paste Electrode-Comparative Study

**DOI:** 10.3390/molecules27072037

**Published:** 2022-03-22

**Authors:** Ewelina Skowron, Kaja Spilarewicz-Stanek, Dariusz Guziejewski, Kamila Koszelska, Radovan Metelka, Sylwia Smarzewska

**Affiliations:** 1 Polfarmex S.A., Jozefow 9, 99-300 Kutno, Poland; ewelina.skowron96@onet.pl; 2 Faculty of Chemistry, Jagiellonian University, Gronostajowa 2, 30-387 Krakow, Poland; kaja.spilarewicz-stanek@uj.edu.pl; 3 Department of Inorganic and Analytical Chemistry, Faculty of Chemistry, University of Lodz, Tamka 12, 91-403 Lodz, Poland; dariusz.guziejewski@chemia.uni.lodz.pl; 4 Department of Analytical Chemistry, Faculty of Chemical Technology, University of Pardubice, Studentska 573, 53210 Pardubice, Czech Republic; radovan.metelka@upce.cz

**Keywords:** square wave voltammetry, paracetamol, graphene, clay, carbon paste electrodes, sensors

## Abstract

The analytical performance of the clay paste electrode and graphene paste electrode was compared using square wave voltammetry (SWV) and cyclic voltammetry (CV). The comparison was made on the basis of a paracetamol (PA) determination on both working electrodes. The influence of pH and SWV parameters was investigated. The linear concentration ranges were found to be 6.0 × 10^−7^–3.0 × 10^−5^ and 2.0 × 10^−6^–8.0 × 10^−5^ mol L^−1^ for clay paste electrode (ClPE) and graphene paste electrode (GrPE), respectively. The detection and quantification limits were calculated as 1.4 × 10^−7^ and 4.7 ×10^−7^ mol L^−1^ for ClPE and 3.7 × 10^−7^ and 1.2 × 10^−6^ mol L^−1^ for GrPE, respectively. Developed methods were successfully applied to pharmaceutical formulations analyses. Scanning electron microscopy and energy-dispersive X-ray spectroscopy were used to characterize ClPE and GrPE surfaces. Clay composition was examined with wavelength dispersive X-ray (WDXRF).

## 1. Introduction

It is well known that chemical sensors are devices that transform chemical information into an analytically useful signal. Among all the chemical sensors reported in the literature, electrochemical sensors are the most attractive because of their remarkable sensitivity, experimental simplicity and low cost. Carbon paste electrodes (CPE) are widely used as working electrodes for the determination of electrochemically active compounds. Paste electrodes have received much attention due to their advantages, such as easily renewable surfaces, inexpensiveness, biocompatibility, and relatively wide potential windows [1,2,3,4,5]. Moreover, the incorporation of modifiers into carbon paste material makes it even more attractive in electroanalytical applications [6,7,8,9,10,11,12]. The performance of chemically modified carbon paste electrodes depends on the properties of the modifier, which affect the selectivity of the electrodes, kinetics of an electrochemical reaction, and sometimes even the electrode reaction product [13,14]. Nowadays, graphene and its derivatives are one of the most popular working electrodes modifiers used in electrochemical studies [15,16,17,18,19]. Graphene is a two-dimensional, sp^2^ hybridized carbon sheet, where atoms are arranged in a honeycomb-shaped lattice [20,21]. Graphene exhibits high thermal conductivity, flexibility, transparency, lightness, and has an extremely high surface area [22,23,24,25]. Because of its interesting properties, graphene has shown great promise in many applications, such as electronics, energy storage and conversion, and electroanalysis or electrocatalysis [24]. Graphene paste electrodes are commonly applied in electrochemistry [26,27,28]. Clays are minerals dominantly made from a colloid fraction of soils, sediments, rocks and water [29]. Grim et al. described clays as an aggregate of minerals and colloidal substances, which are made from a stacking of tetrahedral and octahedral sheets interspersed with a space called the interlayer space. Clay minerals are classified into three families according to the thickness of the sheets, corresponding to a number of tetrahedral and octahedral layers. The gap between sheets can contain water and ions. The tetrahedral sheets are arranged in hexagonal meshes and consist of oxygen tetrahedral surrounding a silicon or aluminum atom. The octahedral sheets are formed by two planes of oxygens-hydroxyl framing broader atoms, such as Al, Fe, Mg and Li [30]. Clay, as an electrode modifier, is a less popular material than, e.g., graphene, although results obtained at such modified electrodes are exceptional [31,32,33,34]. The reason for highly attractive characteristics for electroanalytical purposes are mainly found in the stability of such easily disposable modifiers, both chemical and mechanical, but are also reasoned due to their strong sorptive properties [35]. Therefore, clay and its excellent properties may be revealed in high cation and anion exchange capacity, porosity that imparts changes into the electrical conductivity but also catalytic activity towards electrochemical processes [36,37,38]. The not to be missed advantage of the clay paste electrodes is its extremely low cost, especially when compared to graphene paste electrodes. The aim of this research is to collate differences and similarities in the analytical performance of clay paste and graphene paste electrodes. 

## 2. Results and Discussion 

### 2.1. Preliminary Studies

**Figure 1:**
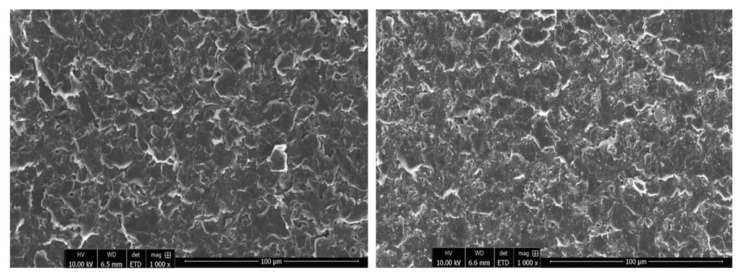
SEM images of graphene paste electrode (**left**) and clay paste electrode (**right**), 1000×.

**Figure 2:**
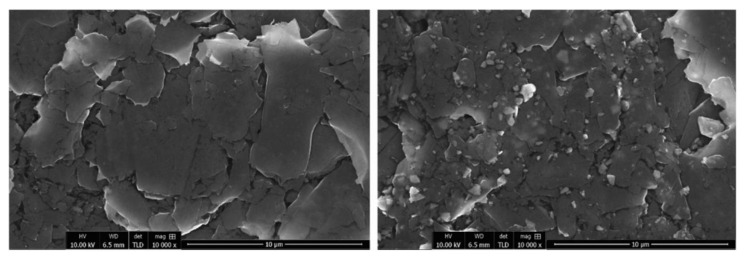
SEM images of graphene paste electrode (**left**) and clay paste electrode (**right**), 10,000×.

Initially, the surfaces of the prepared working electrodes were examined with a scanning electron microscope. In Figure 1, representative SEM images at low magnification of the graphene paste and clay paste coatings are presented. The surface texturing of both coatings is similar. Consecutive SEM images at high magnification are presented in Figure 2, demonstrating the surfaces in detail. Graphene paste exhibits a layered structure, mainly based on graphite plates. However, clay paste has a more heterogeneous structure, showing grains of clay deposited between the graphite plates. The even distribution of grains in the whole film is observed. The investigation of the chemical composition of each type of coatings was also performed with the use of the EDX method (presented in detail in the Supplementary Material). EDX results are in good agreement with data obtained from the WDXRF analysis. 

The electroactive surface of ClPE and GrPE was examined with cyclic voltammetry and a 1.0 mM hexacyanoferrate redox system. The relation between redox peak currents and the square root of the scan rate was found to be linear and the electroactive surface area was calculated with the Randles–Sevcik equation: *I*_p_ = 2.69 × 10^5^ × *n*^3/2^ × *A* × C* × *D*^1/2^ × *υ*^1/2^, where *I*_p_ refers to the peak current, *n* is the number of electrons transferred, *A* is the electroactive surface, *D* is the diffusion coefficient, *υ* is the scan rate and C* is the concentration. *A* values were found to be 1.07 and 1.03 mm^2^ for ClPE and GrPE, respectively.

### 2.2. Voltammetric Studies of Paracetamol

**Figure 3:**
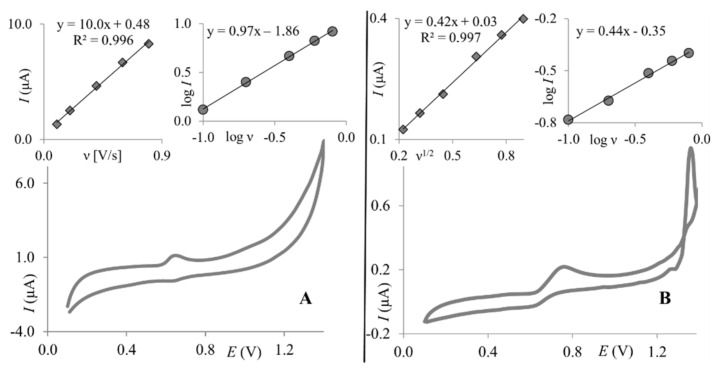
Cyclic voltammograms (scan rate 50 mV × s^−1^) of 1.0 × 10^−5^ mol L^−1^ PA were recorded on ClPE (**A**) and GrPE (**B**). Insets: the relationship between PA peak current and scan rate (**A**-left) or the square root of scan rate (**B**-left); the relationship between the logarithm of PA peak current and logarithm of scan rate (**A**-right, **B**-right).

To compare the electroanalytical performance of ClPE and GrPE, paracetamol was chosen, as its electrochemical behavior was thoroughly investigated by many researchers in various pH using many kinds of working electrodes [14,39,40,41]. This makes it possible to draw up reliable conclusions about observed phenomena. According to the available literature, two possible paths of the oxidation mechanism of paracetamol are known: the one-proton mechanism or the two-proton mechanism, depending on the experimental conditions [39,40,42,43,44]. First, the one-proton mechanism was proposed by Kang et al. (2010) [40]. The latter one, the most commonly observed, involves two electrons and two protons, in which a relatively stable product—N-acetyl-p-benzoquinone-imine (NAPQI) is generated [39,41,42,43]. The occurrence of the follow-up chemical reactions of NAPQI is pH-dependent [39,43]. The electrochemical behavior of paracetamol, on both working electrodes, was first analyzed with cyclic voltammetry. The dependence of scan rate on the PA peak currents was investigated. Figure 3 presents cyclic voltammograms of PA recorded on GrPE and ClPE. As can be seen at both working electrodes, the single anodic peak is visible. The corresponding cathodic signal is much smaller, which is consistent with previous reports discussing paracetamol electrochemical behavior [41].

**Figure 4:**
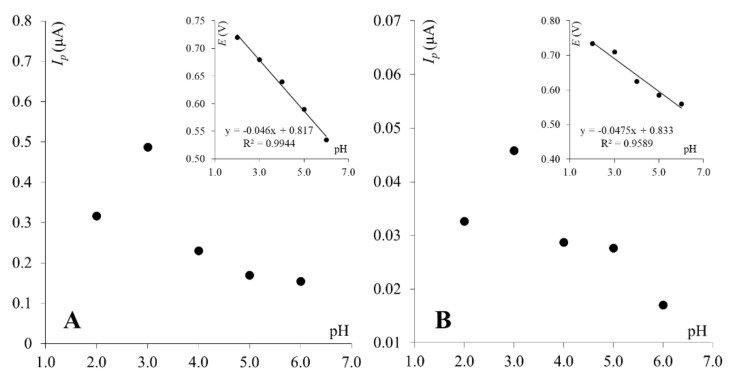
Dependence between PA peak currents and pH of BR buffer for ClPE (**A**) and GrPE (**B**). Insets: dependence between PA peak potentials and pH of BR buffer.

For the clay paste electrode, it was observed that the logarithm of the peak current is linearly proportional to the logarithm of the scan rate (slope = 0.97), indicating an adsorption-controlled electrode reaction (Figure 3A, right inset). This was confirmed by a linear correlation between the PA peak current and the scan rate (Figure 3A, left inset). For graphene paste, electrode adsorption was not observed as the PA peak current was linearly dependent on the square root of the scan rate (Figure 3B, left inset), and the dependence between the logarithm of the peak current and logarithm of the scan rate gave a slope of 0.44 (Figure 3B, right inset), which is close to a theoretical value of 0.5—characteristic for electrode reactions controlled by depolarizer diffusion. Observed differences are in good agreement with previous reports, where it was proven that the paracetamol electrode reaction is dependent on the working electrode surface and material [14,44]. Next, as supporting electrolyte composition may significantly affect the electrochemical behavior of electroactive compounds, the effect of supporting electrolyte pH on PA peak currents was investigated. For this purpose, BR buffers pH 2.0–10.0 were chosen. Similar behavior of paracetamol was observed for both working electrodes. The PA peak current increased with pH to reach the maximum at pH 3.0 on both electrodes and then decreased. At higher pH values (pH ≥ 7), neither the peak currents nor the peak potentials were stable, thus results obtained in pH 7 and higher were not taken into account in further data analyses. Dependences between the PA peak currents and pH of Britton–Robinson buffer obtained on both working electrodes are shown in Figure 4. Based on these dependencies, in order to obtain the highest PA response, BR buffer pH 3.0 was chosen as the optimum supporting electrolyte and used in further studies with both working electrodes. Together, with the peak current, the paracetamol peak potential was also checked, as it frequently is pH-dependent. The PA peak potentials shifted toward less positive values with an increase in the pH for both electrodes. Dependences between the paracetamol peak potentials and pH of the BR buffer were linear for ClPE and GrPE, and could be described using the following equations: *E*_p_ = −0.046 pH + 0.817, and *E*_p_ = −0.048 pH + 0.833, respectively (as can be seen in the inset of Figure 4).

As SWV parameters are interrelated and exert a combined effect on the registered peak currents, in the next experimental step, the influence of SW amplitude, step potential, and frequency on PA signals was studied for ClPE and GrPE. Amplitude (Δ*E*), step potential (Δ*E*_s_), and frequency (*f*) were evaluated in the range of 10–100 mV, 3–21 mV, and 10–100 Hz, respectively. In the case of ClPE, the parabolic dependence of the amplitude on PA signals was observed, the peak current increased from 10 mV to 40 mV, and then decreased. The best results were obtained at 40 mV, and thus this value of amplitude was adopted in subsequent studies. Similarly, a parabolic dependence was observed for frequency analysis, the best-shaped peaks were obtained at 70 Hz. Consequently, 70 Hz was selected for further investigations. Finally, the step potential was evaluated at a whole examined range of step potentials. The PA signals increased with the increase of the Δ*E*_s_ value, but obtained voltammograms were angularly shaped above 7 mV, thus in further studies, a step potential of 7 mV was applied. In the case of GrPE, the effect of amplitude was as follows: the PA peak currents increased from 10 mV to 70 mV, and then a non-linear growth of the peak (plateau) was observed. Therefore, an Δ*E* value of 70 mV was adopted in further studies. The observed PA signals increased with the increasing of *f* and Δ*E*_s_ values at a whole range of frequency and step potential variations. However, a significant deterioration of the peak shape was observed above 30 Hz and 7 mV, thus those values were chosen for subsequent investigations. To summarize, the optimized SW parameters, which were selected with respect to the height and shape of PA signals, were as follows: Δ*E* = 40 mV, *f* = 70 Hz, Δ*E*_s_ = 7 mV and Δ*E* = 70 mV, *f* = 30 Hz, Δ*E*_s_ = 7 mV, for ClPE and GrPE, respectively. 

**Figure 5:**
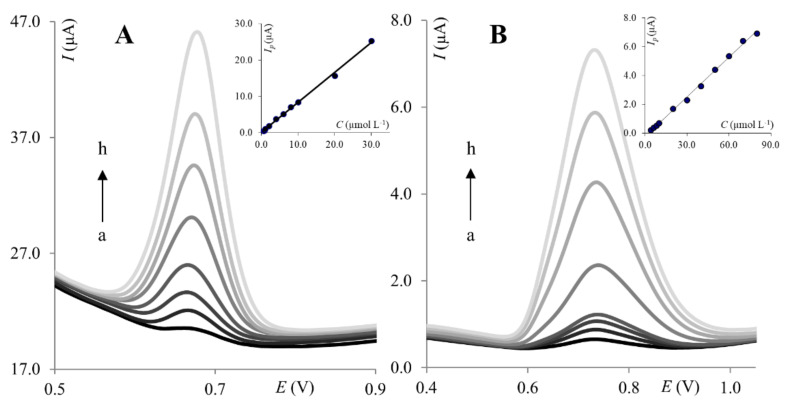
Voltammograms of PA recorded on the ClPE (**A**) and GrPE (**B**). (**A**): (a) 6.0 × 10^−7^ mol L^−1^, (b) 1.0 × 10^−6^ mol L^−1^, (c) 2.0 × 10^−6^ mol L^−1^, (d) 4.0 × 10^−6^ mol L^−1^, (e) 8.0 × 10^−6^ mol L^−1^, (f) 1.0 × 10^−5^ mol L^−1^, (g) 2.0 × 10^−5^ mol L^−1^, (h) 3.0 × 10^−5^ mol L^−1^; (**B**): (a) 4.0 × 10^−6^ mol L^−1^, (b) 6.0 ×10^−6^ mol L^−1^, (c) 8.0 × 10^−6^ mol L^−1^, (d) 1.0 × 10^−5^ mol L^−1^, (e) 2.0 × 10^−5^ mol L^−1^, (f) 4.0 × 10^−5^ mol L^−1^, (g) 6.0 × 10^−5^ mol L^−1^, (h) 8.0 × 10^−5^ mol L^−1^. Insets: Corresponding calibration curves.

Next, the calibration curve was constructed under optimized SWV parameters. The peak current of PA was found to increase linearly with concentration from 6.0 × 10^−7^ to 3.0 × 10^−5^ mol L^−1^ for ClPE (Figure 5A) and from 2.0 × 10^−6^ to 8.0 × 10^−5^ mol L^−1^ for GrPE (Figure 5B).

The linear regression equations can be expressed as:

for ClPE: *I*_p_ (μA) = 0.83 × C_PA_ + 1.56× 10^−7^, (1)

for GrPE: *I*_p_ (μA) = 0.092 × C_PA_ + 2.18 × 10^−7^. (2)

The limit of detection (LOD) and limit of quantification (LOQ) were calculated from the calibration curves as *k*·SD/*b*, where: *k* = 3 for LOD, *k* = 10 for LOQ, SD is the standard deviation of the intercept, *b* is the slope of the calibration curve. The regression parameters for the calibration curves are listed in Table 1. To characterize the reproducibility and stability of the procedure, inter- and intra-day measurements were performed in a solution containing 5.0 × 10^−6^ mol L^−1^ of paracetamol on both working electrodes. The relative standard deviation (RSD) of the voltammetric responses for five repeated detections on the ClPE electrode was 3.0%, whereas, on the GrPE, it was 4.9%. The intra-day reproducibility of the ClPE and GrPE toward PA detection was investigated at the same molar concentration as paracetamol. The relative standard deviation in the peak current for 10 successive assays was 7.8% and 5.2%, respectively.

Although the linear range length is comparable for both electrodes, the linearity observed for ClPE is shifted towards lower concentrations. Therefore, ClPE is a more sensitive sensor towards PA determination. Developed methods were successfully applied for the determination of PA in pharmaceutical formulations. Pharmaceutical formulations containing paracetamol (Paracetamol LG and Paracetamol Polfa) were purchased in a local pharmacy. The PA determination was performed by square wave voltammetry using the standard addition method. As presented in Table 2, no significant differences were observed between the values found by the SWV method and those declared by the producer. However, it is worth noting that results obtained on clay paste electrodes are characterized by a much smaller scatter.

## 3. Materials and Methods

### 3.1. Apparatus

The voltammetric experiments were performed using an EmStat3 potentiostat (PalmSens, Houten, The Netherlands) with an M164 electrode stand (mtm-Anko, Krakow, Poland). All measurements were carried out with a classical three-electrode system and a glass cell of 10 mL volume. A platinum wire and Ag/AgCl (3 mol L^−1^ KCl) were used as the auxiliary and reference electrodes, respectively. Clay paste and graphene paste electrodes were used as the working electrodes. The pH measurements were done using a CP-315 M pH-meter (Elmetron, Zabrze, Poland) with a combined glass electrode. Water was demineralized by means of PURALAB UHQ (made by Elga LabWater, High Wycombe, UK). For microscopic characterization, clay paste and graphene paste were spread onto SEM stubs covered with carbon conductive adhesive tape. SEM images were recorded by a field emission scanning microscope FEI NovaNano SEM 450 using a conventional Everhar–Thornley SE detector (ETD) for 1000× magnification and a thorough lens detector (TLD) in immersion mode for 10,000× magnification. An energy-dispersive X-ray spectroscopy (EDX) was carried out with EDAX TEAM equipped with an EDS Octane Pro detector. Spectrophotometric measurements were made using a Cary 100 Bio UV-Vis spectrophotometer (Agilent). The elemental composition of graphene was analyzed with a Vario MICRO cube: elemental analyzer. The elemental composition of clay powder was examined with the WDXRF spectrometer Panalytical AxiosmAX (lamp Rh-SST–mAX, 4 kW).

### 3.2. Solutions and Materials

All chemicals used in the experiments were of analytical grade and used without further purification. Paracetamol, ferricyanide and all chemicals used for buffers preparation were from Sigma Aldrich (Darmstadt, Germany). Materials used for paste preparation were purchased from Graphene Supermarket (graphite and graphene nanopowder) and Green Club Pharmacy (raw Australian red clay). All water solutions were prepared using distilled and deionized water. Graphene nanopowder was analyzed by combustion analysis (carbon content >99.7%) and UV-VIS spectrometry where one single absorption band at 270 nm was observed (this peak is characteristic for graphene materials and corresponds to the π→π* transition of the C–C bond of the hexagonal carbon ring). The clay elemental composition was tested with a WDXRF spectrometer (page 6 in Supplementary Material). 

### 3.3. Preparation of Working Electrodes

The body of the carbon paste electrode consisted of a Teflon rod (outer diameter of 12 mm) with a horizontal channel (diameter of 4 mm) for the carbon paste filling and metal contact. The paste was prepared by thoroughly (t = 20 min.) hand-mixing 500.0 mg of clay or graphene with 500.0 mg of carbon powder and 0.3 mL of paraffin oil. Before each experiment, the surface of the electrode was refreshed by squeezing out a small portion of paste and polishing it with wet filter paper until a smooth surface was obtained.

### 3.4. Voltammetric Procedure

All voltammetric measurements were carried out at ambient temperature. The general procedure used to obtain square wave (SW) or cyclic (CV) voltammograms was as follows: 10 mL of a supporting electrolyte was transferred to the electrochemical cell. After recording an initial blank, the required volumes of the analyte were added using a micropipette. Then, a sample voltammogram was recorded. For SWV, optimized parameters (amplitude (Δ*E*), frequency (*f*), and step potential (Δ*E*_s_)) were used. 

### 3.5. Analysis of Pharmaceutical Formulations

Commercial tablets of PA (Paracetamol Laboratorium Galenowe and Paracetamol Polfa) were obtained from a local pharmacy (Lodz, Poland). Each tablet contained 500 mg of paracetamol. The pharmaceuticals were prepared by the following procedure. The tablets were accurately weighed and carefully grounded in a mortar. Then, the appropriate amount of the powder was placed in a volumetric flask and filled to volume with distilled and deionized water.

## 4. Conclusions

This paper presents an electroanalytical comparison between the clay paste electrode and the graphene paste electrode. This comparison was made on the basis of a paracetamol determination. For both working electrodes, the supporting electrolyte composition and SW parameters were optimized. In optimal conditions, calibration curves were estimated. Based on the obtained results, it can be concluded that, in general, ClPE and GrPE exhibit similar analytical performance, but clay paste electrodes exhibit a higher sensitivity towards the PA determination, expressed in LOD and LOQ values. Using developed methods, the determination of paracetamol in pharmaceutical formulations was possible with very good recovery on ClPE, as well as on GrPE, however a much smaller data scatter was observed on clay paste electrode. Answering the question “do we really need expensive nanomaterials?” it has to be stated that in comparison to graphene paste electrodes, clay paste electrodes exhibited similar electroactive area and surface morphology, and as mentioned above, improved analytical performance toward paracetamol detection. Moreover, clay paste is a hundred times cheaper than graphene paste. In conclusion, in the authors’ opinion, graphene is undoubtedly an excellent and very promising material but we scientists should always remain open-minded. As shown in this paper, the most expensive and popular materials do not guarantee the best results. Sometimes, ordinary materials serve with a comparable or even higher performance. 

## Figures and Tables

**Table 1:** Regression parameters for determination of PA on ClPE and GrPE, *n* = 3.

Electrode	Clay Paste Electrode	Graphene Paste Electrode
Linear range (mol L^−1^)	6.0 × 10^−7^–3.0 × 10^−5^	2.0 × 10^−6^–8.0 × 10^−5^
Correlation coefficient *R*^2^	0.998	0.997
LOD (mol L^−1^)	1.4 × 10^−7^	3.7 × 10^−7^
LOQ (mol L^−1^)	4.7 ×10^−7^	1.2 × 10^−6^

**Table 2:** Determination of PA in commercial formulations, *n* = 3.

**Clay Paste Electrode**		
	Paracetamol LG	Paracetamol Polfa
Content given (mg)	500.0	500.0
Content found (mg)	480.8 ± 18.0	476.1 ± 23.8
Recovery (%)	96.2	95.2
**Graphene Paste Electrode**		
	Paracetamol LG	Paracetamol Polfa
Content given (mg)	500.0	500.0
Content found (mg)	481.0 ± 47.5	524.6 ± 42.2
Recovery (%)	96.2	104.9

## Data Availability

The data presented in this study are available on request from the corresponding author.

## Supplementary Materials

The following supporting information can be downloaded at: https://www.mdpi.com/article/10.3390/molecules27072037/s1, EDX analysis of clay paste—Analysis no.1; EDX analysis of clay paste—Analysis no.2; EDX analysis of graphene paste; XRF analysis of clay powder.

## Sample Availability

Not available.
