# Supplementary material for: Analytical Performance of Clay Paste Electrode and Graphene Paste Electrode-Comparative Study
Source: Molecules. 2022 Mar 22;27(7):2037. doi: 10.3390/molecules27072037 (PMC9000814; doi:10.3390/molecules27072037)
Supplement: Supplementary file 1 [file molecules-27-02037-s001.zip › molecules-1632658-supplementary.pdf]

EDX analysis of clay paste -ANALYSIS NO. 1

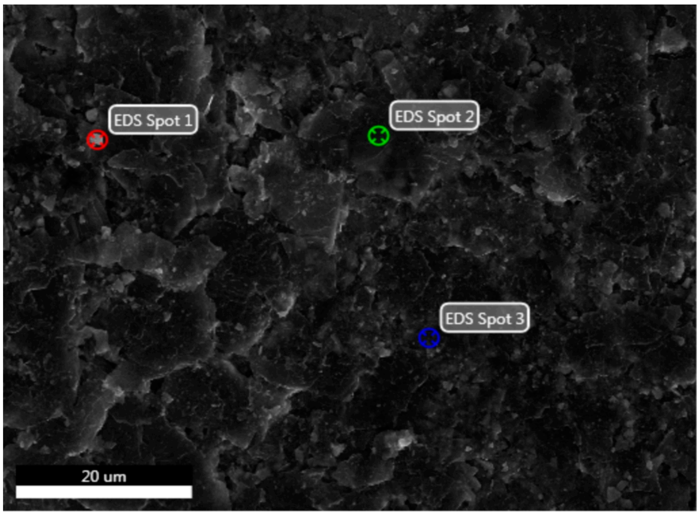

EDS Spot 1

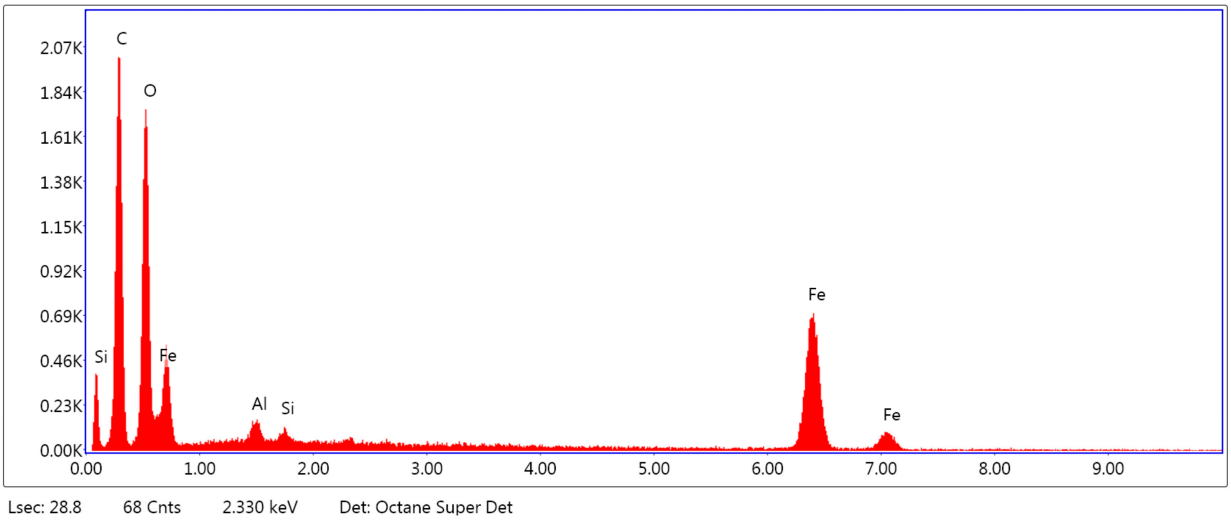

Element	Weight %	Atomic %	Net Int.	Error %	Kratio	Z	R	A	F
C K	39.33	59.82	472.35	8.04	0.1785	1.1138	0.9402	0.4074	1.0000
O K	24.51	27.99	416.23	9.16	0.0811	1.0625	0.9623	0.3112	1.0000
AlK	0.73	0.50	33.31	11.73	0.0045	0.9401	1.0035	0.6458	1.0047
SiK	0.32	0.21	16.41	19.64	0.0023	0.9599	1.0101	0.7560	1.0075
FeK	35.10	11.48	348.74	3.76	0.2924	0.7970	1.0541	1.0103	1.0347

EDS Spot 2

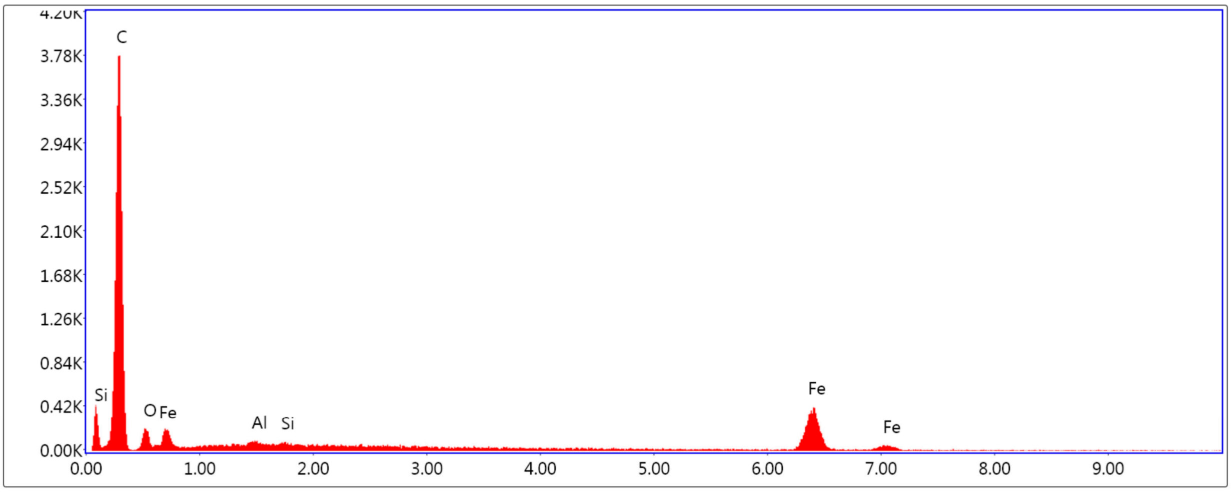

Lsec: 29.3 47 Cnts 2.330 keV Det: Octane Super Det

Element	Weight %	Atomic %	Net Int.	Error %	Kratio	Z	R	A	F
C K	69.94	88.33	861.06	6.34	0.4109	1.0686	0.9610	0.5498	1.0000
O K	5.07	4.81	41.58	13.74	0.0102	1.0181	0.9817	0.1982	1.0000
AlK	0.18	0.10	7.16	57.14	0.0012	0.8991	1.0198	0.7304	1.0050
SiK	0.11	0.06	4.60	63.04	0.0008	0.9179	1.0259	0.8295	1.0080
FeK	24.70	6.71	186.75	4.67	0.1978	0.7600	1.0622	1.0131	1.0399

EDS Spot 3

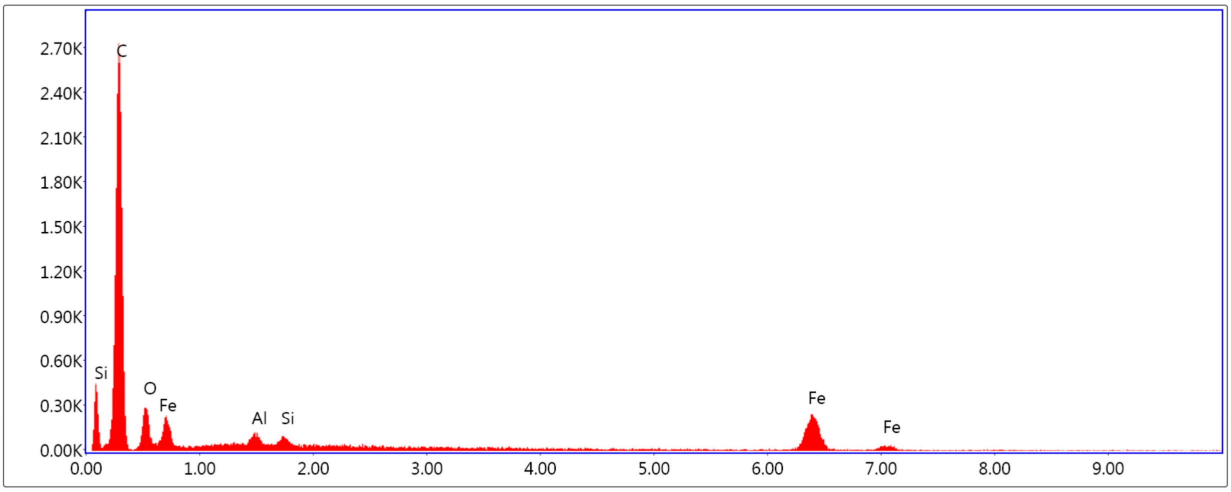

Lsec: 29.3 21 Cnts 2.330 keV Det: Octane Super Det

Element	Weight %	Atomic %	Net Int.	Error %	Kratio	Z	R	A	F
C K	68.20	84.30	615.89	6.47	0.3973	1.0601	0.9664	0.5495	1.0000
O K	10.45	9.69	64.47	12.12	0.0214	1.0096	0.9867	0.2032	1.0000
AlK	0.71	0.39	20.84	14.05	0.0048	0.8911	1.0239	0.7474	1.0050
SiK	0.48	0.25	15.15	19.94	0.0037	0.9095	1.0298	0.8396	1.0077
FeK	20.16	5.36	112.11	5.49	0.1605	0.7523	1.0641	1.0128	1.0447

EDX analysis of clay paste -ANALYSIS NO. 2

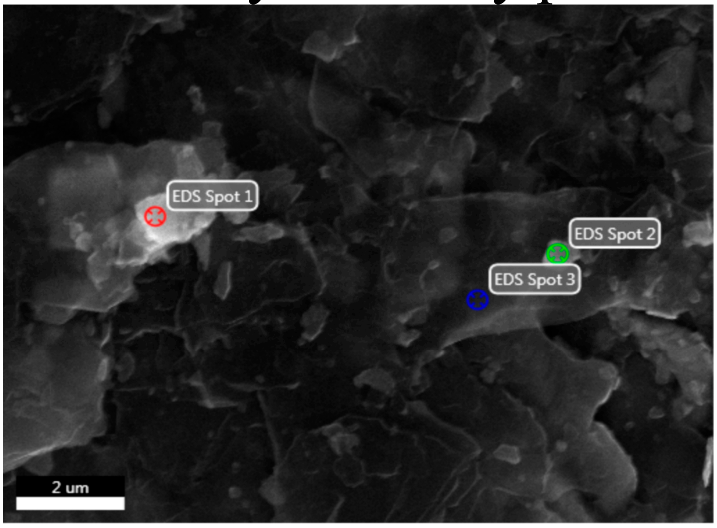

EDS Spot 1

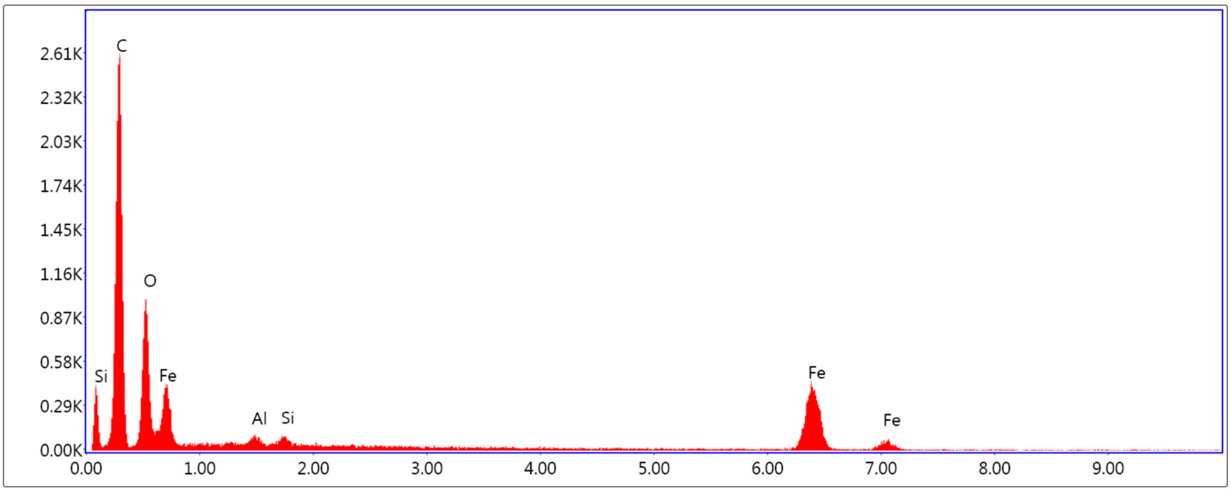

Lsec: 29.2 67 Cnts 1.500 keV Det: Octane Super Det

Element	Weight %	Atomic %	Net Int.	Error %	Kratio	Z	R	A	F
C K	52.28	70.95	583.59	7.25	0.2688	1.0837	0.9548	0.4745	1.0000
O K	20.44	20.83	227.07	10.07	0.0539	1.0329	0.9760	0.2554	1.0000
AlK	0.44	0.27	17.29	17.02	0.0028	0.9126	1.0151	0.6946	1.0048
SiK	0.41	0.24	17.56	18.07	0.0030	0.9317	1.0213	0.7990	1.0075
FeK	26.43	7.72	209.92	4.21	0.2146	0.7719	1.0599	1.0120	1.0393

EDS Spot 2

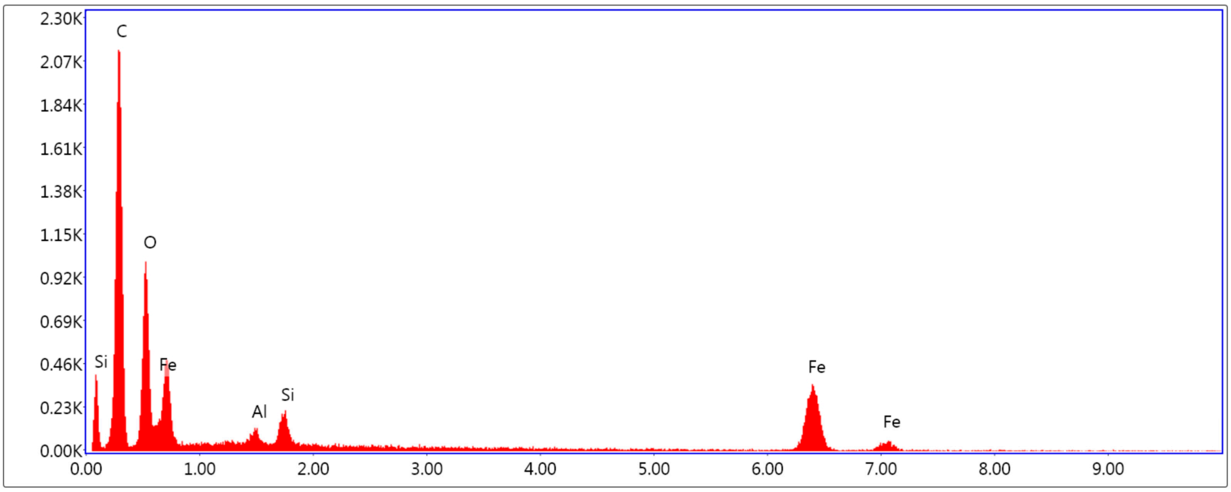

Lsec: 29.2 123 Cnts 1.500 keV Det: Octane Super Det

Element	Weight %	Atomic %	Net Int.	Error %	Kratio	Z	R	A	F
C K	51.15	68.75	487.80	7.48	0.2529	1.0794	0.9576	0.4581	1.0000
O K	22.94	23.14	228.62	10.03	0.0611	1.0286	0.9786	0.2590	1.0000
AlK	0.74	0.44	25.94	12.57	0.0048	0.9085	1.0172	0.7049	1.0051
SiK	1.36	0.78	52.70	8.69	0.0103	0.9275	1.0234	0.8055	1.0072
FeK	23.81	6.88	167.42	4.46	0.1927	0.7679	1.0609	1.0118	1.0417

EDS Spot 3

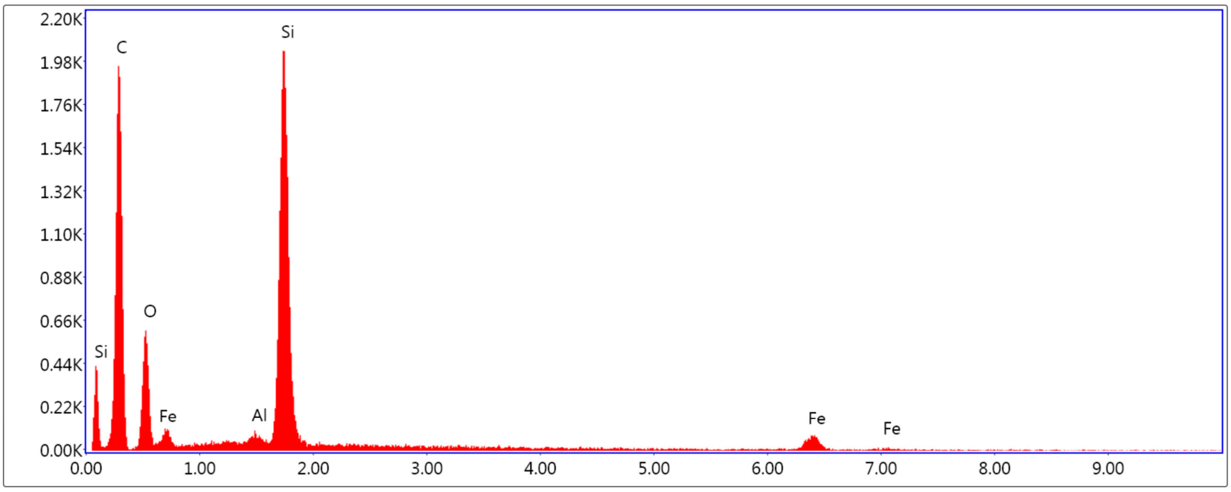

Lsec: 29.2 64 Cnts 1.500 keV Det: Octane Super Det

Element	Weight %	Atomic %	Net Int.	Error %	Kratio	Z	R	A	F
C K	63.19	75.63	435.80	8.75	0.2235	1.0415	0.9791	0.3396	1.0000
O K	17.39	15.63	132.10	11.33	0.0349	0.9908	0.9984	0.2026	1.0000
AlK	0.28	0.15	11.52	21.86	0.0021	0.8729	1.0334	0.8390	1.0150
SiK	14.39	7.37	610.79	3.08	0.1176	0.8908	1.0389	0.9138	1.0045
FeK	4.75	1.22	33.93	12.58	0.0386	0.7348	1.0685	1.0119	1.0947

EDX analysis of graphene paste

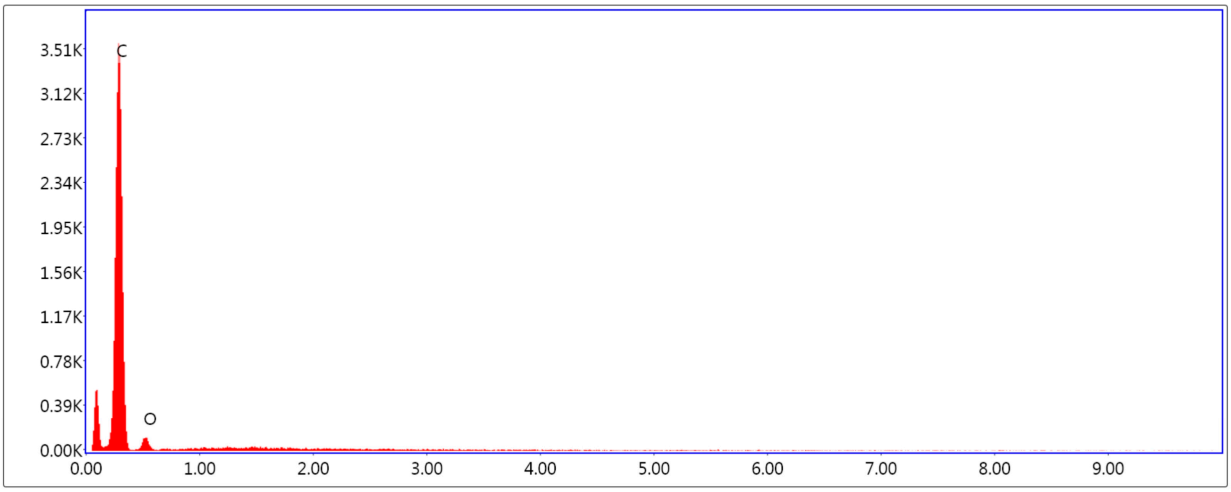

Lsec: 29.5 115 Cnts 0.520 keV Det: Octane Super Det

Element	Weight %	Atomic %	Net Int.	Error %	Kratio	Z	R	A	F
C K	91.06	93.14	831.68	2.63	0.8410	1.0043	0.9983	0.9195	1.0000
O K	8.94	6.86	25.89	14.84	0.0135	0.9540	1.0157	0.1577	1.0000

XRF analysis of clay powder

**Element**	**[mass %]**
Na	0,025 ± 0,003
Mg	0,060 ± 0,004
Al	1,74 ± 0,05
Si	1,26 ± 0,04
P	0,050 ± 0,003
S	0,046 ± 0,003
Cl	0,012 ± 0,002
K	0,027 ± 0,003
Ca	0,119 ± 0,005
Sc	0,0029 ± 0,0007
Ti	0,042 ± 0,004
V	0,013 ± 0,002
Cr	0,0087 ± 0,0013
Mn	0,090 ± 0,005
Fe	65,20 ± 1,20
Zn	0,0037 ± 0,0008
As	0,014 ± 0,002
Zr	0,0018 ± 0,0005
Sb	0,012 ± 0,002
